# Renoprotective Effects of *Allium jesdianum* Against *Cyclophosphamide*: Role of Antioxidant Enzymes and Pro-inflammatory Cytokines in a Murine Model

**DOI:** 10.5812/ijpr-168361

**Published:** 2026-06-28

**Authors:** Alireza Rezaei, Bahareh Sadat Yousefsani, Ameneh Omidi, Kobra Shirani

**Affiliations:** 1 Department of Toxicology, Faculty of Medical Sciences, Tarbiat Modares University, Tehran, Iran; 2 Institute for Studies in Medical History, Persian and Complementary Medicine, Iran University of Medical Sciences, Tehran, Iran; 3 Department of Traditional Medicine, School of Persian Medicine, Iran University of Medical Sciences, Tehran, Iran; 4 Department of Anatomical Sciences, Faculty of Medical Sciences, Tarbiat Modares University, Tehran, Iran

**Keywords:** Allium Jesdianum, Cyclophosphamide, Nephrotoxicity, Antioxidants, Oxidative Stress, Inflammation

## Abstract

**Background:**

Cyclophosphamide (CP), a widely used chemotherapeutic agent, induces significant nephrotoxicity through oxidative stress and inflammation. *Allium jesdianum*, a less-studied wild *Allium* species, has documented antioxidant and anti-inflammatory properties.

**Objectives:**

This study aimed to investigate the renoprotective effects of *A. jesdianum* extract (AJE) against CP-induced kidney injury, focusing on its simultaneous modulation of oxidative and inflammatory pathways.

**Methods:**

Mice were randomly divided into four groups: Control, AJE only, CP only, and AJE + CP. Treatments were administered for 14 days. Kidney function, oxidative stress markers, inflammatory cytokines, and histopathological changes were evaluated.

**Results:**

CP administration significantly increased serum urea and creatinine levels, enhanced lipid peroxidation, and reduced antioxidant enzyme activities. Histopathological analysis revealed marked renal damage. Pretreatment with AJE significantly attenuated these alterations by restoring renal function markers (P < 0.01), reducing oxidative stress (P < 0.001), and enhancing antioxidant defenses (P < 0.001). Compared with the CP-only group, AJE also significantly reduced levels of pro-inflammatory cytokines (P < 0.001).

**Conclusions:**

AJE exerts significant protective effects against CP-induced nephrotoxicity through dual antioxidant and anti-inflammatory mechanisms.

## 1. Background

Renal toxicity induced by cyclophosphamide (CP) remains a significant clinical challenge in chemotherapy and often results in dose-limiting nephrotoxicity characterized by oxidative stress, inflammation, and tissue damage (1, 2). Despite its therapeutic efficacy, CP administration promotes reactive oxygen species generation, depletes antioxidant defenses, and activates inflammatory pathways, ultimately compromising renal function (3, 4).

Current protective strategies against CP-induced nephrotoxicity are limited and often rely on synthetic antioxidants or supportive care, which may not fully address the multifactorial nature of renal injury. Therefore, there is growing interest in identifying natural adjuvants with dual antioxidative and anti-inflammatory properties to enhance renal resilience during chemotherapy.

Among natural candidates, plants of the *Allium* genus, particularly less-studied species such as *Allium jesdianum*, show promise because of their rich content of bioactive compounds, including flavonoids and organosulfur derivatives, which are known to modulate oxidative and inflammatory responses (5, 6). Although previous studies have demonstrated the general protective effects of common *Allium* species, such as garlic and onion, against drug-induced toxicities, research on *A. jesdianum* remains scarce, particularly regarding its simultaneous effects on oxidative stress and cytokine-mediated inflammation in CP-induced renal injury (7, 8). Beyond the *Allium* genus, other medicinal plants, such as *Pimpinella anisum*, have also been shown to exert protective effects by modulating inflammatory responses and oxidative stress, further supporting the potential of plant-derived natural products as multi-target therapeutic agents (9).

## 2. Objectives

Most existing investigations have focused on isolated pathways, either oxidative stress or inflammation, rather than adopting an integrated approach to comprehensively evaluate renoprotection. This focus represents a critical knowledge gap in understanding how multi-target natural agents such as *A. jesdianum* can mitigate complex chemotherapy-related organ damage. Therefore, the present study aimed to investigate the protective potential of *A. jesdianum* extract (AJE) against CP-induced nephrotoxicity by simultaneously assessing key markers of oxidative stress and inflammation in a murine model. This integrative approach was designed to provide novel mechanistic insights and to evaluate *A. jesdianum* as a promising natural adjunct for renal protection during CP-based therapies.

## 3. Methods

### 3.1. Plant Sample Preparation

*Allium jesdianum* was collected in spring 2021 from a uniform wild population in the Bakhtiari Mountains, Iran, and taxonomically authenticated by an expert botanist at Jundishapur University, Ahvaz, where a voucher specimen (Code: A-0138) was deposited. To ensure batch consistency and reproducibility, the entire quantity of plant material used in this study was harvested in a single season, dried under identical conditions, and processed as a single homogenized batch. One hundred grams of dried whole-plant powder was extracted with 300 mL of 80:20 (v/v) ethanol-to-water solvent by standardized maceration at room temperature for 72 hours. The extract was concentrated under reduced pressure using a rotary evaporator at 40°C, freeze-dried, and further dried in a vacuum oven at 40°C for 5 days to obtain a stable solid residue. The entire extract batch was stored in airtight, light-protected containers at 4°C until use to prevent degradation. Total phenolic compounds were quantified using the Folin-Ciocalteu spectrophotometric assay with gallic acid as the standard, and total flavonoid content was determined using a colorimetric aluminum chloride assay with quercetin as the standard.

### 3.2. Animals

Six- to eight-week-old NMRI mice weighing 19 to 21 g were purchased from the Faculty of Medical Sciences at Tarbiat Modares University in Tehran, Iran. Before the experimental procedures, the mice were housed in polystyrene cages (5 animals per cage) and acclimated to the laboratory environment for 1 week. During this period, the mice were maintained under standard conditions, including a temperature range of 20 - 22°C, a relative humidity of approximately 35%, and a 12-hour light/dark cycle. The mice had free access to a standard laboratory diet and drinking water throughout the acclimation period. All animal procedures were performed in accordance with ethical standards and were approved by the Ethical Committee of Iran University of Medical Sciences (Approval No. IR.IUMS.AEC.1403.041). No animals were excluded from the analysis; all mice completed the study without premature death or severe morbidity.

### 3.3. Treatment Protocols

NMRI mice were randomly divided into four groups, each consisting of 5 animals. The first group served as the negative control (NC) and received normal saline orally for 14 days. The second group received an oral dose of 200 mg/kg AJE daily for 14 consecutive days. The third group received the same dose of AJE (200 mg/kg orally) for 14 days, in addition to intraperitoneal (IP) injections of CP at 20 mg/kg during the last 5 days of the period. The fourth group served as the positive control and received IP injections of CP at 20 mg/kg for 5 days. These protocols were designed based on previous studies.

### 3.4. Body and Relative Organ Weight

To evaluate weight changes, the body weight of each mouse was recorded at baseline (day 1) before the initial treatment. On day 14, 2 hours after the final dose administration, the animals were anesthetized with ketamine (100 mg/kg) and xylazine (10 mg/kg). After anesthesia, the mice were euthanized by spinal cord severance. Kidney weights were recorded manually. Relative organ weights were calculated as the ratio of kidney weight to total body weight (organ weight/body weight).

### 3.5. Serum Collection and Tissue Preparation

Before tissue collection, blood samples were collected individually from each mouse via cardiac puncture into sterilized, dry centrifuge tubes. The samples were left to coagulate at 37°C for 10 minutes and then centrifuged at 2500 rpm for 10 minutes to separate the serum. The serum was sent to a laboratory for biochemical analysis to evaluate a range of parameters. Next, the kidneys were carefully dissected and rinsed with an ice-cold isotonic saline solution to remove blood and debris. Tissues designated for histopathological evaluation, lipid peroxidation, and antioxidant enzyme assessments were prepared accordingly.

### 3.6. Histopathological Studies

For histopathology, kidney tissue was fixed in 10% neutral-buffered formalin for 24 - 48 hours. The fixed samples were then processed, embedded in paraffin, and sectioned into 5-μm slices. The sections were stained with hematoxylin and eosin and examined under a light microscope to evaluate morphological changes indicative of tissue injury or pathology.

Histopathological scoring was performed by an independent histologist who was blinded to group allocation. Five randomly selected, non-overlapping fields per kidney section (×400 magnification) were evaluated. A semi-quantitative scoring system from 0 to 4 was applied based on the percentage of cortical damage: 0 = normal; 0.5 = small focal damage; 1 = < 10% damage; 2 = 10 - 25% damage; 3 = 25 - 75% damage; and 4 = > 75% damage. Higher scores indicated greater damage. Table 1 provides the detailed scoring criteria.

**Table 1.** Histopathological Scoring of Kidney Tissue

Pattern of Renal Necrosis	Score
**Normal morphometry**	0
**Small focal damage area**	0.5
**Cortical damage zone (%)**	
< 10	1
10 - 25	2
25 - 75	3
> 75	4

### 3.7. Biochemical Parameters

Kidney function was assessed by measuring serum creatinine and urea levels (10).

### 3.8. Kidney Tissue Lipid Peroxidation Assay

Malondialdehyde (MDA) levels, a marker of lipid peroxidation, were quantified using the thiobarbituric acid (TBA) assay (Navand Salamat). According to the manufacturer's instructions, tissue homogenates were combined with TBA reagent to generate colored products. After centrifugation, the absorbance of the supernatants was measured at 532 nm, and MDA concentrations were calculated from a standard curve (11).

### 3.9. Antioxidant Enzyme Assessments

Antioxidant enzyme activities, including superoxide dismutase (SOD), catalase (CAT), and glutathione peroxidase (GSH-Px), were measured in kidney tissue using commercial assay kits according to the manufacturers' protocols (Navand Salamat) (12).

### 3.10. Inflammatory Cytokine Parameters

Inflammatory cytokines, including tissue levels of interleukin 6 (IL-6), interleukin 1 beta (IL-1β), and tumor necrosis factor alpha (TNF-α), were measured using commercial ELISA kits according to the manufacturers' protocols (Karmania Pars Gene) (13).

### 3.11. Statistical Analyses

All results were expressed as mean ± standard deviation. Data among groups were analyzed by one-way analysis of variance, followed by Tukey's honest significant difference post hoc test. A value of P < 0.05 was considered statistically significant. All statistical analyses were performed using Prism statistical software version 20.

## 4. Results

### 4.1. Total Flavonoid and Phenolic Compound Content

The extract contained 157.6 mg of total phenolic compounds and 114.7 mg of total flavonoid compounds per gram of dry extract.

### 4.2. Body and Relative Organ Weight

No significant differences in body weight were observed among the experimental groups before treatment. Cyclophosphamide administration at 20 mg/kg/day resulted in a statistically significant reduction in body weight compared with the control group (P < 0.05). No significant effects on kidney weight or relative kidney weight were detected in any treatment group. No mortality was observed in any group (Table 2).

**Table 2.** The Protective Effect of AJE on Body Weight and Kidney Weight in Mice ^a^

Parameters	NC	AJE	AJE + CP	CP
**Body weight (g)**				
Before treatment	28 ± 2	27.5 ± 2.5	28.1 ± 2	28.1 ± 2.8
After treatment	34.5 ± 3.5	34 ± 1.5	33 ± 3	29 ± 3.2 ^b^
**Kidney weight (mg)**	333 ± 6.1	329 ± 4.3	325 ± 6.8	315 ± 5.8
**Relative kidney weight (%BW)**	1.01%	0.98%	0.98%	1.09%

^a^ Data are presented as mean ± SD (n = 5). Abbreviations: AJE, *Allium jesdianum* extract; CP, cyclophosphamide; NC, negative control; %BW, percentage of body weight.

^b^ P < 0.05 indicates a significant difference compared with the control group.

### 4.3. Histopathological Examination

**Figure 1.**
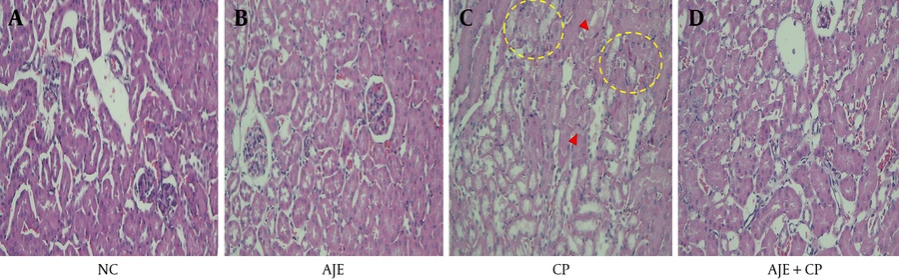
The impact of AJE on kidney tissue histopathology. Representative images of kidney tissue stained with hematoxylin and eosin and examined under a microscope (magnification: ×400). Red arrowheads show cellular vacuolization, and yellow dashed circles indicate areas of disintegration in the renal architecture. Abbreviations: AJE, *Allium jesdianum* extract; CP, cyclophosphamide; NC, negative control.

As shown in Figure 1A and B, histopathological examination of the kidney revealed that the NC group and the group treated with AJE alone exhibited normal renal tissue architecture, with histopathological scores of 0 in both groups (Table 1). In contrast, the CP-treated group exhibited marked renal damage, characterized by structural and morphological disruptions and significant vacuolization (Figure 1C), and therefore received a high renal necrosis score of 3, corresponding to 25 - 75% cortical damage according to the scoring criteria in Table 1. Pretreatment with AJE before CP administration restored normal renal morphology and reduced vacuolization (Figure 1D), which was quantitatively reflected by a significantly lower necrosis score of 1 (< 10% cortical damage) in the AJE + CP group (Table 1) (14).

### 4.4. Biochemical Parameters

As indicated in Table 3, renal toxicity was observed after CP intraperitoneal injection and was characterized by significant increases in serum urea and creatinine levels (P < 0.001 and P < 0.01, respectively) compared with the normal control group. Pretreatment with AJE significantly alleviated the increases in serum urea and creatinine levels (P < 0.001 and P < 0.01, respectively).

**Table 3.** The Effect of AJE on Kidney Function Markers in Mice ^a^

Parameters	NC	AJE	AJE + CP	CP
**Urea (mg/dL)**	32 ± 8	38 ± 11	41 ± 5 ^b^	60 ± 7 ^c^
**Creatinine (mg/dL)**	0.30 ± 0.09	0.31 ± 0.05	0.35 ± 0.03 ^d^	0.48 ± 0.02 ^e^

^a^ Values are expressed as mean ± SD. Statistical analyses were conducted using the Tukey-Kramer test. Abbreviations: AJE, *Allium jesdianum* extract; CP, cyclophosphamide; NC, negative control.

^b^ P < 0.001 indicate significant differences compared with the CP group .

^c^ P < 0.001 indicate significant differences relative to the normal saline group.

^d^ P < 0.01 indicate significant differences compared with the CP group.

^e^ P < 0.01 indicate significant differences relative to the normal saline group.

### 4.5. Lipid Peroxidation Assay

**Figure 2.**
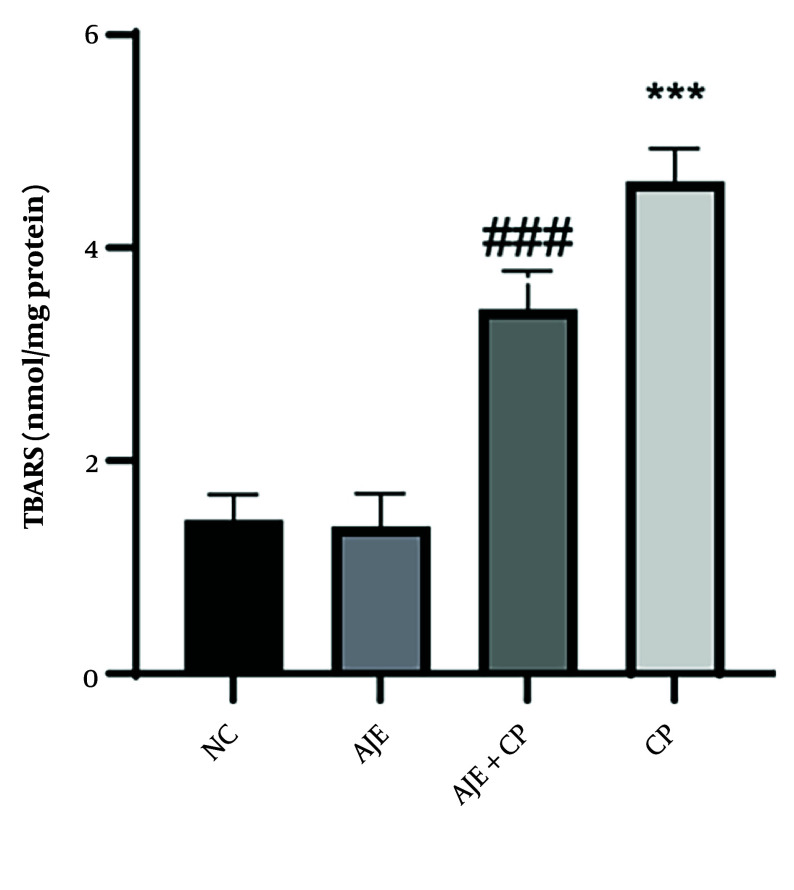
The effect of AJE on lipid peroxidation in mouse kidney tissue. Data are presented as mean ± standard deviation (n = 5). Statistical analyses were conducted using the Tukey-Kramer test. *** P < 0.001 indicates a significant difference relative to the normal saline group. ### P < 0.001 indicates a significant difference compared with the CP group. Abbreviations: AJE, *Allium jesdianum* extract; CP, cyclophosphamide; NC, negative control; TBARS, thiobarbituric acid reactive substances.

Cyclophosphamide injection led to a significant increase in kidney TBARS levels compared with the NC group (P < 0.001). AJE pretreatment reduced TBARS levels compared with the CP-only group (P < 0.001) (Figure 2).

### 4.6. Antioxidant Enzyme Assessments

#### 4.6.1. Superoxide Dismutase Activity

**Figure 3.**
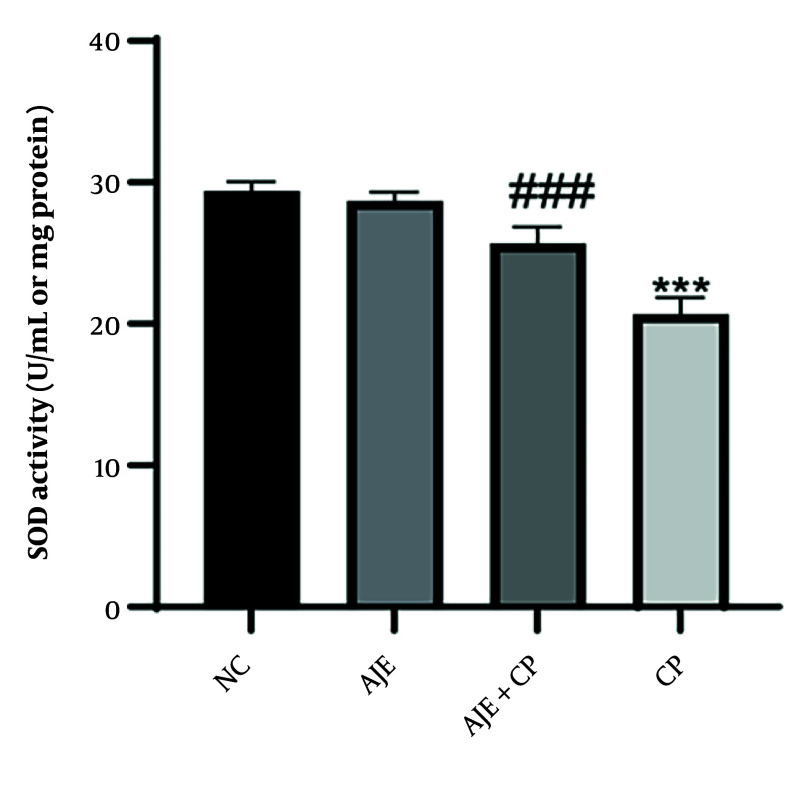
The effect of AJE on superoxide dismutase activity in mouse kidney tissue. Data are presented as mean ± standard deviation (n = 5). Statistical analyses were conducted using the Tukey-Kramer test. *** P < 0.001 indicates a significant difference relative to the normal saline group. ### P < 0.001 indicates a significant difference compared with the CP group. Abbreviations: AJE, *Allium jesdianum* extract; CP, cyclophosphamide; NC, negative control; SOD, superoxide dismutase.

SOD activity was decreased in the CP group compared with the NC group (P < 0.001). AJE administration increased SOD activity compared with the CP group (P < 0.001) (Figure 3).

#### 4.6.2. Catalase Activity

**Figure 4.**
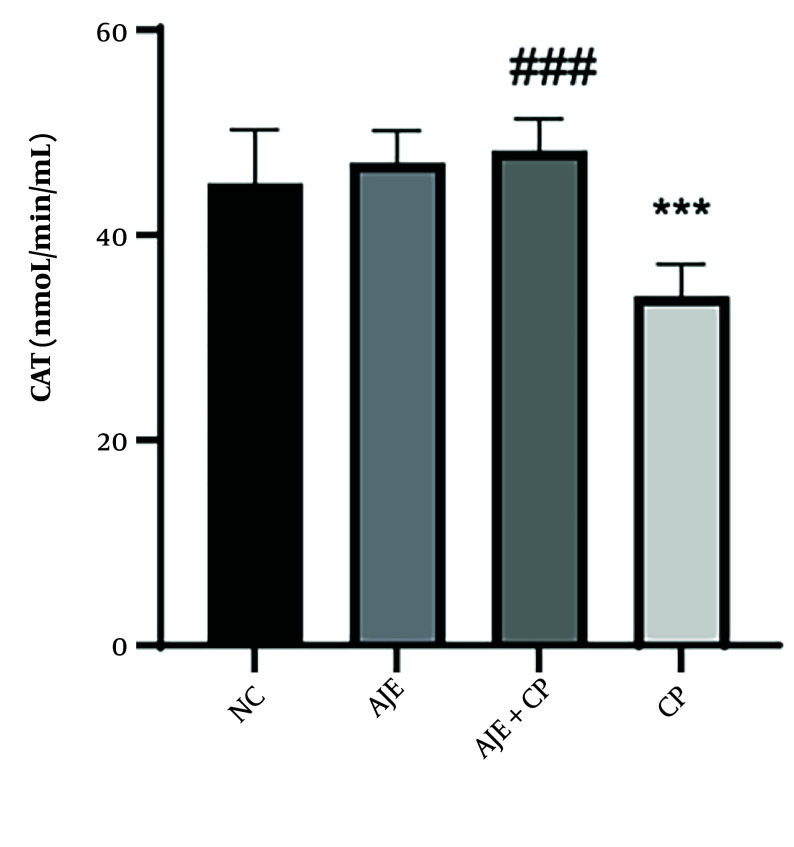
The effect of AJE on catalase activity in mouse kidney tissue. Data are presented as mean ± standard deviation (n = 5). Statistical analyses were conducted using the Tukey-Kramer test. *** P < 0.001 indicates a significant difference relative to the normal saline group. ### P < 0.001 indicates a significant difference compared with the CP group. Abbreviations: AJE, *Allium jesdianum* extract; CAT, catalase; CP, cyclophosphamide; NC, negative control.

CAT activity was reduced in the CP group compared with the NC group (P < 0.001). AJE treatment elevated CAT activity compared with the CP group (P < 0.001) (Figure 4).

#### 4.6.3. Glutathione Peroxidase Activity

**Figure 5.**
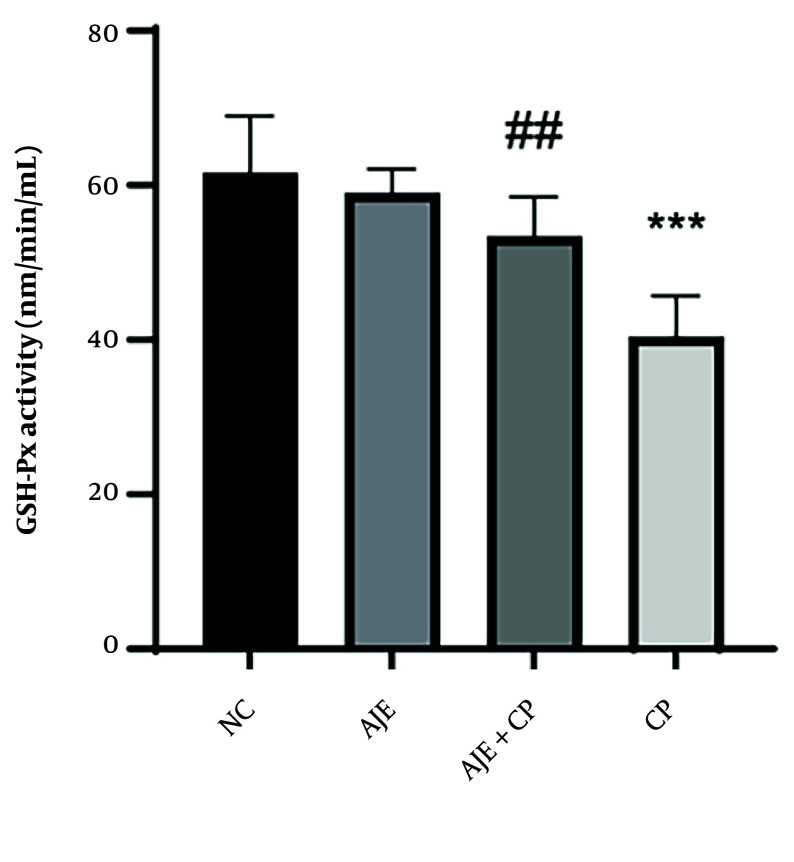
The effect of AJE on glutathione peroxidase activity in mouse kidney tissue. Data are presented as mean ± standard deviation (n = 5). Statistical analyses were conducted using the Tukey-Kramer test. *** P < 0.001 indicates a significant difference relative to the normal saline group. ## P < 0.01 indicates a significant difference compared with the CP group. Abbreviations: AJE, *Allium jesdianum* extract; CP, cyclophosphamide; GSH-Px, glutathione peroxidase; NC, negative control.

GSH-Px activity was decreased in the CP group compared with the NC group (P < 0.001). AJE administration restored GSH-Px activity compared with the CP group (P < 0.01) (Figure 5).

### 4.7. Inflammatory Cytokine Parameters

#### 4.7.1. Interleukin 6

**Figure 6.**
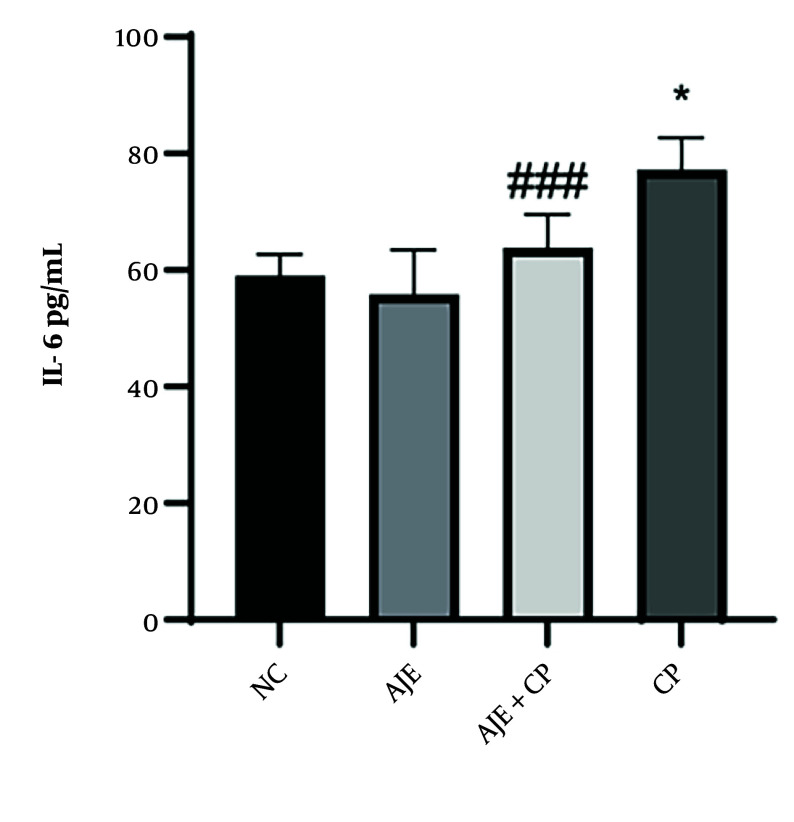
The effect of AJE on interleukin 6 activity in mouse kidney tissue. Data are presented as mean ± standard deviation (n = 5). Statistical analyses were conducted using the Tukey-Kramer test. * P < 0.05 indicates a significant difference relative to the normal saline group. ### P < 0.001 indicates a significant difference compared with the CP group. Abbreviations: AJE, *Allium jesdianum* extract; CP, cyclophosphamide; IL-6, interleukin 6; NC, negative control.

As illustrated in Figure 6, the NC group showed baseline levels with significantly lower IL-6 concentrations than the treatment groups. Cyclophosphamide administration increased IL-6 levels in kidney tissue compared with the NC group (P < 0.05). AJE pretreatment reduced IL-6 levels compared with the CP-only group (P < 0.001) (Figure 6).

#### 4.7.2. Interleukin 1 Beta

**Figure 7.**
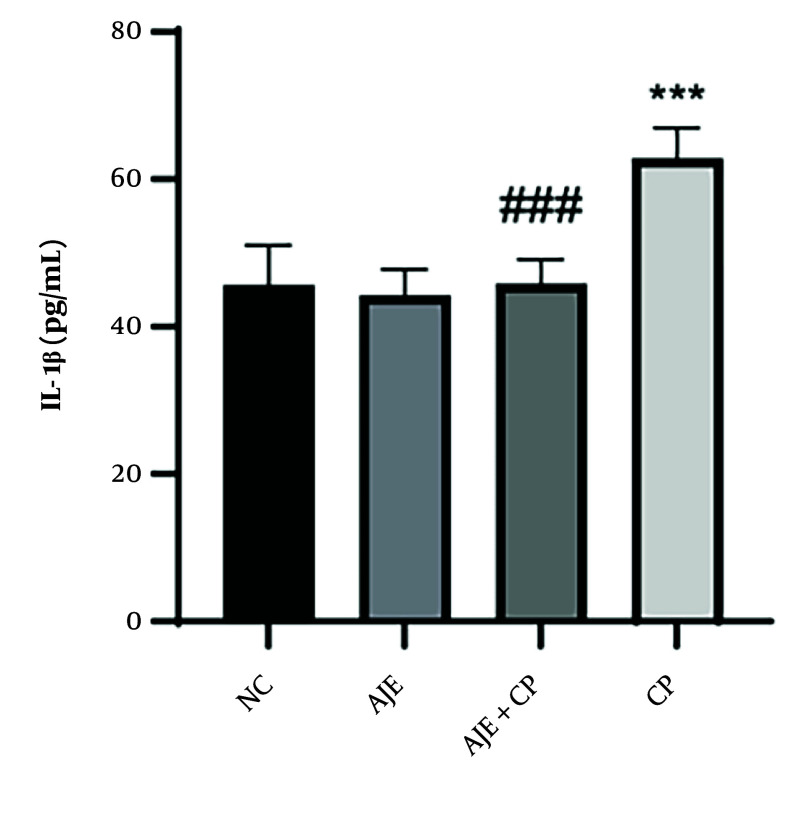
The effect of AJE on interleukin 1 beta activity in mouse kidney tissue. Data are presented as mean ± standard deviation (n = 5). Statistical analyses were conducted using the Tukey-Kramer test. *** P < 0.001 indicates a significant difference relative to the normal saline group. ### P < 0.001 indicates a significant difference compared with the CP group. Abbreviations: AJE, *Allium jesdianum* extract; CP, cyclophosphamide; IL-1β, interleukin 1 beta; NC, negative control.

Cyclophosphamide administration increased IL-1β levels compared with the NC group (P < 0.001). AJE pretreatment reduced IL-1β levels compared with the CP-only group (P < 0.001) (Figure 7).

#### 4.7.3. Tumor Necrosis Factor Alpha

**Figure 8.**
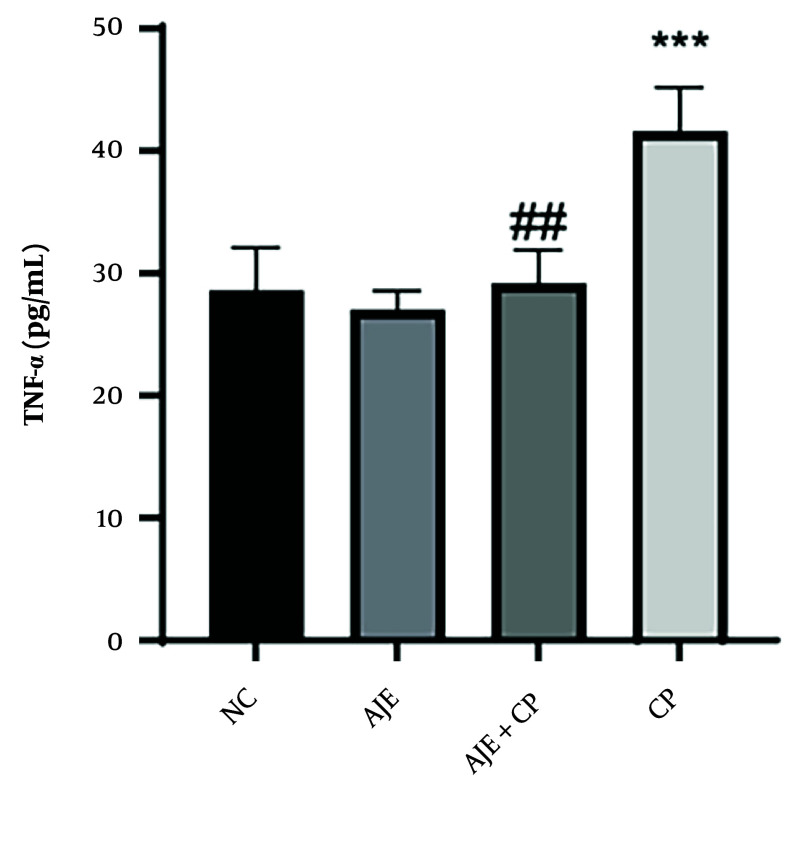
The effect of AJE on tumor necrosis factor alpha activity in mouse kidney tissue. Data are presented as mean ± standard deviation (n = 5). Statistical analyses were conducted using the Tukey-Kramer test. *** P < 0.001 indicates a significant difference relative to the normal saline group. ## P < 0.01 indicates a significant difference compared with the CP group. Abbreviations: AJE, *Allium jesdianum* extract; CP, cyclophosphamide; NC, negative control; TNF-α, tumor necrosis factor alpha.

Cyclophosphamide administration increased TNF-α levels compared with the NC group (P < 0.001). AJE pretreatment reduced TNF-α levels compared with the CP-only group (P < 0.01) (Figure 8).

## 5. Discussion

Cyclophosphamide remains a cornerstone chemotherapeutic agent for various malignancies; however, its clinical utility is often compromised by dose-limiting toxicity mediated by oxidative stress, inflammation, and apoptotic pathways. In this regard, antioxidant-based strategies have shown promising protective effects against CP-induced organ toxicity, supporting the broader relevance of antioxidant agents or supplements as adjunctive therapies (15).

This study demonstrates that AJE confers significant renoprotection in a murine model of CP-induced kidney injury through concurrent modulation of oxidative and inflammatory mediators.

Consistent with prior reports, CP administration in this model induced renal dysfunction, as evidenced by elevated serum urea and creatinine levels (P < 0.001), increased MDA levels (P < 0.001), and depletion of SOD, CAT, and GSH-Px (P < 0.001). Histopathological alterations, including vacuolization and tubular damage, further confirmed CP-induced nephropathy (16, 17). AJE pretreatment counteracted these changes, restoring biochemical parameters and renal histoarchitecture.

The novelty of these findings lies in the dual-pathway efficacy of AJE. Unlike many previous studies that focused solely on either the antioxidant or anti-inflammatory properties of *Allium* species, this work provides integrated evidence that AJE simultaneously enhances endogenous antioxidant defenses (SOD, CAT, and GSH-Px) and suppresses pro-inflammatory cytokines (IL-6, IL-1β, and TNF-α). These findings suggest that AJE operates through multi-target mechanisms, likely mediated by its phenolic and flavonoid profile, which are known to scavenge reactive oxygen species and modulate signaling cascades such as the NF-κB and Nrf2/ARE pathways (18, 19). For instance, quercetin, a prominent flavonoid in *Allium* species, has been shown to inhibit NF-κB nuclear translocation and suppress the activation of downstream pro-inflammatory genes, thereby attenuating cytokine production in renal tissues (20, 21). Furthermore, organosulfur compounds present in AJE may enhance the activity of the nuclear factor erythroid 2-related factor 2 (NRF2)/antioxidant response element pathway, upregulating antioxidant gene expression and reinforcing cellular resistance to oxidative insult (22, 23). Such coordinated modulation of both oxidative and inflammatory axes underscores the potential of AJE as a holistic renoprotective agent in the context of CP-induced nephrotoxicity.

Our results align with and extend previous findings on the nephroprotective effects of *Allium* species. For example, *Allium sativum* (garlic) has demonstrated similar antioxidative and anti-inflammatory effects in drug-induced nephrotoxicity models (24). Co-administration of shallot (*Allium ascalonicum* L.) extract with cyclosporine A ameliorated the adverse effects of cyclosporine A on kidney function, oxidative stress parameters, and histopathological alterations. These findings demonstrate the renoprotective capacity of shallot extract against cyclosporine A-induced nephrotoxicity, with evidence suggesting that its antioxidant activity plays a key role in mediating this protective effect (25). Administration of the hydroalcoholic extract derived from *A. jesdianum* has been shown to mitigate carbon tetrachloride (CCl_4_)-induced nephrotoxicity. This renoprotective effect is likely mediated through the potent antioxidant properties of the extract, which contribute to scavenging free radicals generated by CCl_4_ exposure (26). However, much of the existing literature on wild *Allium* species, including *A. jesdianum*, has been limited to phytochemical screening or single-pathway investigations. To our knowledge, this is the first report to delineate the concurrent modulation of both oxidative stress and cytokine-mediated inflammation by *A. jesdianum* in CP-induced renal injury, thereby filling a notable gap in the phytopharmacology of less-characterized *Allium* species.

From a clinical perspective, the use of natural adjuvants such as AJE could help mitigate chemotherapy-associated renal damage without interfering with the chemotherapeutic efficacy of CP. The observed multi-target activity supports its potential as a complementary renoprotective agent, possibly reducing the need for dose reduction or discontinuation of CP in vulnerable patients. Future studies should explore whether AJE also protects against CP-induced toxicity in other organs, such as the bladder and heart, to evaluate its broader protective profile (27, 28).

### 5.1. Limitations and Future Directions

Several limitations must be acknowledged. First, the sample size (n = 5 per group) may affect statistical power. Second, dose-response evaluations were not performed. Third, molecular pathway analyses, such as western blotting for NF-κB and Nrf2, were not conducted. Fourth, compound-specific identification via HPLC/LC-MS was not feasible because of resource limitations; therefore, the exact bioactive constituents remain unidentified. Future research should address these gaps through dose-ranging studies, protein expression analyses, and bioactivity-guided fractionation.

### 5.2. Conclusions

In summary, AJE demonstrates significant protective effects against CP-induced nephrotoxicity through integrated antioxidative and anti-inflammatory mechanisms. These findings not only underscore the therapeutic potential of understudied *Allium* species but also highlight the value of multi-target natural products in managing chemotherapy-induced organ injury. Despite its limitations, this study provides a robust foundation for further mechanistic and translational investigations into AJE as a feasible adjunct to CP-based regimens.

## Data Availability

The dataset presented in the study is available on request from the corresponding author during submission or after publication.
